# Leiomyomatosis peritonealis disseminata: Three case reports

**DOI:** 10.1097/MD.0000000000022633

**Published:** 2020-10-09

**Authors:** Lei Yang, Na Liu, Yun Liu

**Affiliations:** Department of Obstetrics and Gynecology, Beijing Friendship Hospital, Capital University of Medical Sciences, Beijing, China.

**Keywords:** case reports, laparoscopic myomectomy, leiomyomatosis peritonealis disseminata

## Abstract

**Rationale:**

Leiomyomatosis peritonealis disseminata (LPD) is a rare benign lesion primarily consisting of smooth muscle cells, which mostly affects premenopausal females. Here, we reported 3 females with LPD (age, 40–48 years) admitted for pelvic masses.

**Patient concerns:**

All 3 LPD cases received laparoscopic uterine fibroid morcellation at 3, 8, and 14 years ago, respectively. Two cases were admitted for pelvic masses. One case was admitted for recurrent fibroids with pollakiuria.

**Diagnoses:**

LPD was considered in 2 cases preoperation according to imaging examination, and one of them received ultrasound-guided biopsy of the lesion in the right lobe of the liver. One case was considered as recurrent fibroids preoperation. After surgery, all cases were pathologically diagnosed as LPD consisting of benign smooth muscle cells.

**Interventions:**

A total abdominal hysterectomy, salpingo-oophorectomy, and debulking was performed for all 3 cases. Intraoperative exploration revealed that the fibroids distributed in the mesentery (3 cases), broad ligament (1 case), omentum (1 case), liver (1 case), and rectus abdominis (1 case).

**Outcomes:**

No recurrence was found during postoperative following-up (5–12 months).

**Lesions:**

Preoperative diagnosis of LPD is presented as a challenge due to unspecific clinical manifestations. Its diagnosis mainly depends on histopathologic evaluation. Surgery still is the primary treatment for LPD. For patients without reproductive desire, total abdominal hysterectomy, salpingo-oophorectomy, and debulking can be performed, and the affected tissue should be removed as much as possible based on the risk assessment.

## 1 Introduction

Leiomyomatosis peritonealis disseminata (LPD) is characterized by dissemination and proliferation of peritoneal and subperitoneal lesions primarily originated from smooth muscle cells. LPD is more common in premenopausal women. Although benign in nature, LPD may degenerate into peritoneal leiomyosarcoma.^[1,2]^ So far, no more than 200 cases of LPD have been reported. However, the published case reports concerning LPD following laparoscopic myomectomy are increasing. Preoperative diagnosis of LPD is presented as a challenge due to unspecific clinical manifestations and its clinical resemblance with peritoneal carcinomatosis or metastatic lesions. Herein, we reported 3 cases of LPD from Beijing Friendship Hospital, Capital Medical University between June 2018 and May 2019.

## 2 Case report

### 2.1 Case 1

**Figure 1:**
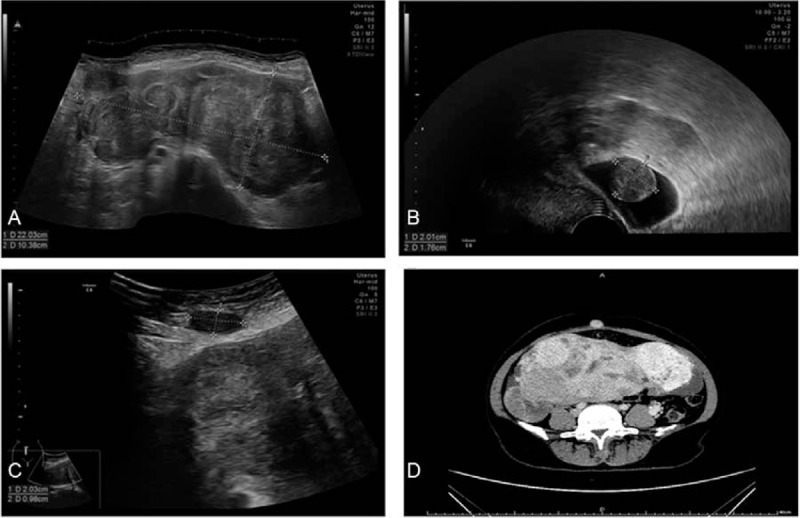
Ultrasound and CT images of Case 1. A, Transvaginal ultrasound showed multiple hypoechoic nodules in uterus, and the uterus lost its normal shape. B, Transvaginal ultrasound showed hypoechoic nodules in uterine rectal area. C, Transabdominal ultrasound showed hypoechoic nodule in paraumbilical abdominal wall. D, Enhanced CT scan revealed irregular soft tissue mass in the lower abdomen and nodules in abdominal wall below the umbilicus.

**Figure 2:**
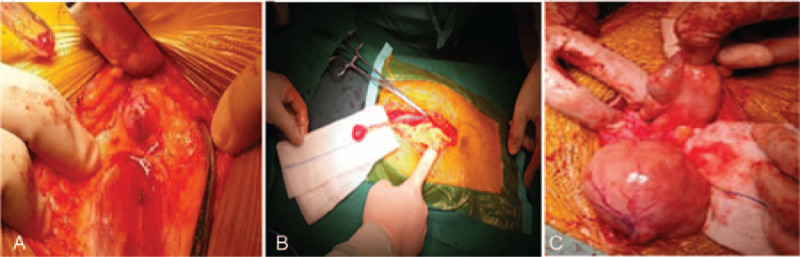
Intraoperative images of Case 1. Leiomyomatosis peritonealis disseminata nodules involving (A) the surface of anterior rectus sheath and near the umbilicus, (B) the inferior margin of omentum, and (C) the beginning of the ascending colon laparoscopic visualization.

A 40-year-old female was admitted for treatment of pelvic, abdominal, and abdominal wall masses. The patient had a past history of 2 pregnancies and 1 birth. Fourteen years ago, she underwent laparoscopic myomectomy. Ten years ago, she was diagnosed as recurrent uterine fibroids (3 cm) during health examination. Thereafter, she did not receive any treatment for asymptomatic and progressively increased fibroids. After admission, gynecological examination revealed that the size of uterus increased as large as that of 5 months pregnancy. Transvaginal ultrasound showed multiple hypoechoic nodules in the anterior and posterior wall of the uterus (largest size of 14.1×11.3×9.1 cm) (Fig. 1A) and hypoechoic nodules in utero rectal fossa (Fig. 1B) and paraumbilical abdominal wall (Fig. 1C). The CT scan revealed irregular soft tissue mass in the lower abdomen and nodules in abdominal wall below the umbilicus (Fig. 1D). Laboratory tests showed a CA125 level of 80.4 mIU/mL. Following preoperative evaluation, laparotomy was scheduled. During the operation, a tumescent nodule (1.5 cm in diameter) was detected on the surface of anterior rectus sheath and near the umbilicus (Fig. 2A). Then, another nodule (2 cm in diameter) was detected on the inferior margin of greater omentum (Fig. 2B). Moreover, multiple tumescent nodules were found on surface of uterus, with the maximum diameter of 14 cm. No obvious abnormalities were observed on the bilateral uterine appendages. Furthermore, a nodule was detected on the right peritoneal surface of the upper rectum, with a diameter of about 2 cm. Additionally, a nodule was found at the beginning of the ascending colon, with a diameter of 8 cm (Fig. 2C). Finally, hystero-salpingo-oophorectomy and pelvic and abdominal mass resection were performed. The histopathologic analysis showed that there were benign smooth muscle cells in the tissue sample from the excisional nodules. No recurrence was found during the first postoperative year.

### 2.2 Case 2

**Figure 3:**
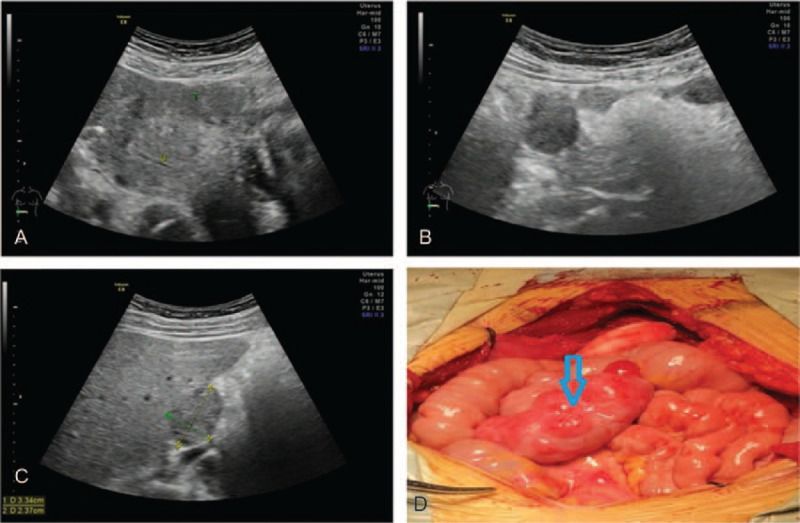
Ultrasound and intraoperative images of Case 2. A, B, Ultrasound showed multiple masses in the pelvic and abdomen. C, Ultrasound showed a hypoechoic nodule on lower edge of the right hepatic lobe. D, Multiple bead-like hard nodules on the surface of small intestine near to ileocecal junction.

A 40-year-old female was admitted for pelvic and abdominal masses. She had a past history of 4 pregnancies and 2 births. Three years ago, she underwent laparoscopic myomectomy, which was converted to open surgery due to major haemorrhage. Histopathologic evaluation of the excisional tissues revealed a tumor consisting of smooth muscle cells. Six months ago, she received ultrasound examination, which showed multiple masses in the pelvic and abdomen (Fig. 3A and B), mainly in the uterus and surrounding peritoneum, mesenteric space, and the lower edge of the right hepatic lobe (Fig. 3C). Then ultrasound-guided biopsy of the lesion in the right lobe of the liver was performed and a leiomyoma was revealed. Physical examination revealed that the size of uterus increased as large as that of 8 weeks pregnancy and that the uterus adhered to the anterior wall. The level of CA125 was normal. During exploratory laparotomy, about 50 mL bloody fluid was observed in the abdominal cavity. And multiple tumescent nodules were found on surface of uterus, with the maximum diameter of about 5 cm. There was dense adhesion between uterus and the posterior wall of the bladder. Multiple bead-like hard nodules ranging from about 0.5 to 3 cm in diameter were detected on the surface of a 1 m of small intestine near to ileocecal junction (Fig. 3D). A hystero-salpingo-oophorectomy was performed. The histopathologic analysis of the tissue sample from the excisional nodules revealed multiple leiomyomas. No malignant cell was found in peritoneal lavage smear. No recurrence was found during the 5 months postoperative follow-up.

### 2.3 Case 3

**Figure 4:**
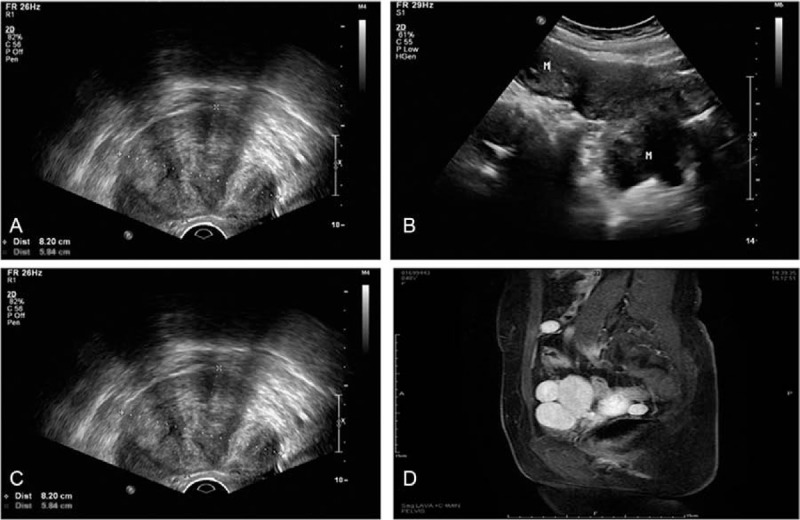
Ultrasound and MRI images of Case 3. A, B, Ultrasound showed multiple hypoechoic nodules in the uterus. C, Ultrasound showed a hypoechoic nodule in uterine rectal fossa, suggesting subserosal fibroids. D, Contrast-enhanced magnetic resonance imaging suggested multiple uterine fibroids and subserosal fibroids.

A 48-year-old female was admitted for pollakiuria lasting for 6 months. She had no past history of pregnancy and child birth. Eight years ago, she underwent laparoscopic myomectomy in our institution. Three years ago, multiple uterine fibroids were found again. Thereafter, fibroids were presented as progressive enlargement during regular evaluations. After this admission, gynecological examination revealed that the size of the uterus increased as large as that of 14 weeks pregnancy. Ultrasound showed multiple hypoechoic nodules in the anterior and posterior wall of the uterus (the maximum diameter of 8 cm) (Fig. 4A, B, and C). MRI suggested multiple uterine fibroids and subserosal fibroids (posterior uterine abnormal signal) (Fig. 4D). Laboratory tests showed a CA125 level of 99.8 mIU/mL. She was planned to receive surgery for recurrent fibroids. However, during laparoscopic exploration, multiple myoma-like nodules were scattered in the surface of the right iliac ligament, anterior lobe of left broad ligament, anterior parietal peritoneum abdominal and mesentery, with varied diameters ranging from 2 cm to 6 cm. The hystero-salpingo-oophorectomy and pelvic lesions resection were performed. The histopathologic analysis of the tissue sample from the excisional nodules revealed LPDs. No recurrence was found during the postoperative 3-month follow-up.

## 3 Discussion

LPD, first introduced by Wilson et al in 1952, is an exceedingly rare but generally benign lesion caused by uterine fibroids or tissue disseminated and implanted on the surface of the greater omentum, mesentery, and colorectus, which is also known as parasitic leiomyoma.^[3]^ Currently, no more than 200 cases of LPD have been revealed worldwide. Its etiology and pathophysiology remain unclear. It has been proposed that LPD could be caused by metaplasia of mesenchymal cells of the peritoneum and, in susceptible women, residual myoma in the abdominal cavity might contribute to the development of LPD.^[4]^ Metaplasia and differentiation from mesenchymal stem cells into smooth muscle cells may be promoted by estrogen exposure.^[5]^ Therefore, LPD is often considered a premenopausal benign disease. However, a few cases of LPD have been diagnosed in menopausal women or with malignant transformation.^[1,2]^

Iatrogenic origin subsequent to laparoscopic surgery has been suggested as another main theory of the etiology and pathophysiology of LPD in setting of development and widely application of laparoscopic uterine fibroids morcellation.^[6]^ In 1997, Ostrzenski^[7]^ reported the first case of uterine leiomyoma particle growing in an abdominal-wall incision after laparoscopic retrieval. There has been an increasing number of reports on iatrogenic LPD following laparoscopic surgery in past decades.^[6]^ In this study, all 3 cases received laparoscopic uterine fibroid morcellation 3, 8, and 14 years ago, respectively. Therefore, laparoscopic uterine fibroid morcellation may be an important factor for secondary disseminating of fibroids.

The preoperative diagnosis of LPD is a challenge because patients are often asymptomatic or presented as untypical symptoms such as abdominal pain and discomfort, and irregular vaginal bleeding. Erenel et al^[8]^ described 53 patients with LPD including 28 (53%) cases presenting as abdominal pain, 13 asymptomatic patients and others with pelvic palpable mass. In this study, all 3 cases had a primary presentation of pelvic and abdomen masses, and 1 case also had pollakiuria. The LPD diagnosis in Case 2 was confirmed by preoperative liver biopsy pathological evaluation. Although LPD is a benign disorder, it has imaging characteristics of disseminating in pelvic and abdomen consistent with malignancy. Therefore, in coexist of elevated serum level of CA125 and disseminating implantation, it is difficult to differentiate LPD from malignancy. Preoperative diagnosis of LPD depends on comprehensive evaluation of medical history and imaging studies, especially for patients with a history of laparoscopic uterine fibroid morcellation. Ultrasonography, CT, and MRI are among the most effective diagnostic methods, but they are not of great help in the differential diagnosis of malignancies. In fact, only histopathologic examination on preoperative biopsy tissues can confirm the diagnosis of LPD.

There are very few cases of LPD that will undergo malignant transformation. Currently, only about 10 cases with malignant transformation have been reported.^[2,9–16]^ The risk factors for malignant transformation of LPD include no history of oral contraceptives, no history of pregnancy, no history of uterine leiomyoma, no expression of estrogen and progesterone receptors in leiomyomas nodules, and recurrence within 1 year after initial treatment.^[9]^ For patients with high-risk factors, vaginal ultrasound and MRI should be scheduled to conduct a regular follow-up.

There are no clear guidelines regarding the treatment of LPD. Recently, it has been proposed that surgical therapy should be individualized according to the patient's age, symptoms, child-bearing requirement, and past treatments.^[17]^ In principle, the scope of surgery should be as far as possible to remove all tumor nodules. For women with no reproductive desire, a more extensive surgical approach with total abdominal hysterectomy, salpingo-oophorectomy, and debulking may be the best alternative. However, whether the fibroids on the surface of the intestinal wall should be primarily resected is still debatable. We conducted a literature research and found only1 case underwent emergency surgery due to ileus caused by 5 cm fibroid nodules on the surface of the intestinal wall.^[18]^ No other adverse outcome associated with metastatic myoma nodules on intestinal wall was reported. In Case 2, we found multiple bead-like fibroid nodules in a 1 m segment of small intestine, and the diameter of the nodules was about 0.5 to 3 cm. The risk of intestine injury is extremely high in setting of resection of fibroid nodules. Considering that this patient without child-bearing requirements, only hysterectomy and salpingo-oophorectomy was performed, without fibroid nodules resection. No recurrence was found during the 5 months postoperative follow-up. In addition, patients suspected of LPD should be informed prior to surgery that an extensive and complicated surgery may be performed. Moreover, susceptible dissemination location of LPD nodules should also be carefully explored to minimize the risk of postoperative recurrence.

LPD is also closely associated with estrogen. Therefore, it has been suggested that gonadotropin-releasing hormone injection, aromatase inhibitor, or selective progesterone receptor modulator can be used for conservative or primary treatment of young women with reproductive desire and prevention of postoperative recurrence.^[19–22]^ The efficacy and safety of such drugs in LPD patients need to be confirmed by further clinical case studies due to lack of clinical evidence.

In conclusion, LPD is a rare benign clinical disorder with risk of recurrence. The uterine fibroids patients with a history of uterine surgery are susceptible to LPD. Therefore, for patients without reproductive desire, total abdominal hysterectomy, salpingo-oophorectomy, and debulking can be performed, and the affected tissue should be removed as much as possible based on the risk assessment.

## Author contributions

**Data curation:** Lei Yang.

**Formal analysis:** Na Liu.

**Methodology:** Yun Liu.

**Resources:** Yun Liu.

**Writing – original draft:** Lei Yang, Na Liu.

**Writing – review & editing:** Yun Liu.

## Abbreviations

Abbreviation: LPD = leiomyomatosis peritonealis disseminata.
